# Human Breast Milk Extracellular Vesicles Mitigate Endothelial Dysfunction

**DOI:** 10.3390/nu17182953

**Published:** 2025-09-13

**Authors:** Young-Eun Cho, Shaoshuai Chen, Keith Crouch, Damon Shutt, Justin W. Kaufman, Brajesh K. Singh

**Affiliations:** 1 College of Nursing, The University of Iowa, Iowa City, IA 52242, USA; shaoshuai-chen@uiowa.edu (S.C.); keith-crouch@uiowa.edu (K.C.); damon-shutt@uiowa.edu (D.S.); 2 Stead Family Department of Pediatrics, Carver College of Medicine, The University of Iowa, Iowa City, IA 52242, USA; justin-kaufman@uiowa.edu

**Keywords:** human breast milk extracellular vesicles, exosomes, endothelial dysfunction, inflammation, oxidative stress

## Abstract

**Background**: Endothelial cell (EC) dysfunction is an early sign of compromised vascular integrity and is associated with various cardiovascular diseases (CVDs). Activation of Toll-like receptor 4 (TLR4) plays a central role in this process. Extracellular vesicles (EVs) derived from milk have known anti-inflammatory properties, particularly in suppressing TLR4 activation. This study investigates the therapeutic potential of human breast milk-derived EVs (HBM-EVs) in mitigating EC dysfunction related to CVDs. **Methods**: HBM-EVs were isolated from the breast milk of healthy nursing mothers using ultracentrifugation. HBM-EVs were applied to lipopolysaccharide (LPS)-treated human umbilical vein endothelial cells (HUVECs), and inflammatory marker expression was assessed through qPCR and Western blotting. Mitochondrial oxidative stress was measured using MitoSOX. The effects of HBM-EVs were further evaluated in ex vivo studies using mesenteric arteries from diet-induced obese mice. Additionally, the effect of HBM-EVs on angiogenesis was tested via a wound closure assay. **Results**: In HUVECs, pre-treatment with HBM-EVs inhibited LPS-induced expression of inflammatory markers, including IL-6 and VCAM-1, as well as the phosphorylation of NFκB. Additionally, HBM-EVs reduced LPS-induced mitochondrial oxidative stress. In animal studies, HBM-EV treatment restored EC-dependent vasorelaxation in mesenteric arteries from diet-induced obese mice. Furthermore, HBM-EVs enhanced EC migration, leading to improved wound closure in HUVECs. **Conclusion**: This study demonstrates the therapeutic potential of HBM-EVs in alleviating EC dysfunction, offering a promising new approach to the treatment of CVDs. Future research will focus on identifying the specific cargo of HBM-EVs and further exploring their therapeutic mechanisms in endothelial dysfunction.

## 1. Introduction

Endothelial cells (ECs) play a crucial role in maintaining cardiovascular homeostasis by controlling vascular reactivity, angiogenesis, permeability, and blood clotting [1]. Changes in EC function mark the onset of early cardiovascular disease (CVD) stages, consequently contributing to the progression of diverse cardiovascular conditions such as atherosclerosis, hypertension, and coronary artery diseases [2,3]. Increased inflammation is a hallmark of altered EC function. It contributes to increased oxidative stress and reduced vessel relaxation. It also contributes to impaired therapeutic angiogenesis in ECs, thereby preventing the stimulation of new blood vessel growth and improving blood flow in ischemic tissues. Toll-like receptor 4 (TLR4) signaling plays a critical role in impairing these EC functions. TLR4 is highly expressed in microvascular ECs, and activated TLR4 is involved in promoting inflammatory response, leading to the development and progression of CVDs [4,5,6]. Therefore, inhibiting TLR4 is considered a therapeutic strategy for CVD treatment.

Extracellular vesicles (EVs) are cell-derived, lipid bilayer-enclosed nanosized particles (30–150 nm in diameter) that carry a diverse repertoire of functional molecules—including microRNAs (miRNAs), messenger RNAs (mRNAs), long noncoding RNAs, DNA fragments, proteins, and lipids [7,8,9]. They are secreted by almost all cell types and have been detected in a wide range of biological fluids, including serum, urine, saliva, and breast milk. Among these fluids, human milk is particularly enriched in EVs, which are increasingly recognized as key modulators of neonatal immune and metabolic development [10,11]. Beyond their developmental role, a growing body of evidence has highlighted the therapeutic potential of milk-derived EVs, particularly their potent anti-inflammatory and immunoregulatory effects within the intestinal tract. In both in vivo and in vitro models of inflammatory bowel disease, milk EVs have been shown to attenuate mucosal inflammation, reduce epithelial apoptosis, and promote regeneration of intestinal architecture and tight junction integrity [12,13,14]. These protective effects have been mechanistically linked to inhibition of TLR4. Specifically, treatment with milk EVs reduces TLR4 expression, which in turn lowers levels of inflammatory markers such as NFκB and the NLRP3 inflammasome, as well as oxidative stress [13,14,15]. Importantly, the therapeutic relevance of milk EVs may extend beyond the gut. Several animal studies have demonstrated that milk EVs remain stable in biological fluids and retain functional integrity after systemic administration [16,17,18]. Biodistribution analyses have detected milk EVs in distant organs—including the liver, spleen, lung, and brain—, suggesting milk EVs can modulate biological processes beyond the intestinal environment and may serve as systemically active therapeutic agents.

Despite growing interest in EV-based therapies, most milk EV studies to date have primarily focused on intestinal outcomes, leaving their effects on other physiological systems largely unexplored. Given that milk EVs remain stable in circulation and exert anti-inflammatory effects through TLR4 inhibition, it is plausible that they may also confer protective effects against endothelial dysfunction, a key contributor to cardiovascular diseases. Endothelial cells (ECs) form a critical interface between the bloodstream and underlying tissues, and they are highly responsive to inflammatory cues, and TLR4 signaling plays a central role in promoting vascular inflammation, all hallmark features of EC dysfunction. In this study, we investigated the potential of human breast milk-derived EVs to alleviate EC dysfunction. Our findings offer new insight into the therapeutic properties of milk EVs and provide foundational evidence supporting their application in preventing or treating cardiovascular conditions linked to endothelial inflammation.

## 2. Methods

**Breast Milk Collection**. Human breast milk was collected as described previously published study [19]. Briefly, we recruited healthy nursing mothers who were ≥18 years old and had delivered a full-term (37–40 weeks) singleton newborn within the prior six months (*n* = 15). Mothers with chronic conditions that might affect body weight changes, such as chronic diabetes or thyroid diseases, were excluded. After electronic informed consent was obtained, a breast milk sample collection kit, including milk collection pouches and detailed instructions, was sent to the participant’s home by the 3rd week of postpartum. Per study instructions, participants self-collected 50 mL of breast milk in the morning before breakfast at approximately 3–5 weeks postpartum, using either a breast pump or hand expression. Milk samples were frozen in their home freezer. Within 48 h of sample collection, samples were returned to the lab and stored at −80 °C until use. All procedures were conducted under an approved IRB protocol by the University of Iowa Human Subject Office/Institutional Review Board (IRB #202005237, approved 4 December 2020). Written informed consent was obtained from all subjects involved in the study. To avoid potential effects caused by the postpartum period and maternal BMI, milk from mothers whose pre-pregnancy BMI was either <25 (normal weight) and a postpartum day less than 40 days was used in this experiment. Fresh human breast milk samples were collected and transported to the laboratory (~30 min) in a cooler bag with frozen ice packs, maintaining the temperature below 4 °C. Upon arrival, samples were immediately stored at −80 °C until further processing. For EV isolation, samples were thawed once on ice and processed immediately to minimize EV degradation. After isolation, purified HBM-EVs were aliquoted and stored at −80 °C until further use.

**Breast Milk Extracellular Vesicle Isolation and Characterization**. Human breast milk EVs (HBM-EVs) were isolated using ultracentrifugation, as described previously [19]. Before EV isolation, frozen milk samples were thawed once on ice to minimize freeze–thaw cycles. To obtain skimmed milk, samples were centrifuged at 3000× *g* for 15 min, repeated 2–3 times, to sequentially remove fat and cellular debris. In this study, casein depletion was not performed because our aim was to analyze the total HBM-EV population. For EV isolation, the clarified skimmed milk supernatant was ultracentrifuged at 100,000× *g* for 2 h, and the resulting pellet was washed once with sterile PBS and subjected to a second ultracentrifugation under the same conditions. The final EV pellet was resuspended in sterile PBS and rotated overnight at 4 °C before downstream analyses. The concentration and the size of HBM-EVs were analyzed using NanoSight NS300 (Malvern Panalytical, Malvern, UK). Also, we tested surface epitopes of HBM-EVs using a MACSPlex Exosome Kit (Miltenyi Biotec, Bergisch Gladbach, Germany), which can detect up to 37 surface epitopes of EVs [19]. In addition, the expression level of EV markers including CD9, CD81 and CD63 was measured using NanoView R100 (NanoView Biosciences, Brighton, MA, USA). The concentration of HBM-EV was measured with Qubit™ Protein and Protein Broad Range (BR) Assay Kits (Thermo Fisher Scientific, Waltham, MA, USA).

**Cell Viability Assay.** HUVECs were seeded in 96-well plates and cultured for 12 h, followed by treatment with various concentrations of HBM-EVs (0–400 µg/mL) for 48 h. MTT solution (5 mg/mL in PBS; Promega, Madison, WI, USA) was then added to each well to reach a final concentration of 0.5 mg/mL. After 4 h of incubation at 37 °C, the medium was removed and 150 μL of DMSO was added to dissolve the formazan crystals. The absorbance was measured at 570 nm using a microplate reader (SpectraMax iD5, Molecular Devices, LLC., San Jose, CA, USA)

**HUVECs Culture and HBM-EV Treatment.** Human umbilical vein endothelial cells (HUVECs) were purchased from ATCC (Manassas, VA, USA) and cultured in EGM-2 Endothelial Cell Growth Medium-2 BulletKit (Lonza, Basel, Switzerland) at 37 °C with 5% CO_2_ and 95% air conditions until passage 5 [20,21]. The same culture conditions were maintained throughout the other experiments involving HUVECs. When cells reached 70–80% confluence, HBM-EVs or PBS (vehicle control) were treated for 24 h, followed by LPS treatment for another 24 h. The treatment time and concentration of HBM-EVs (48 h and 50 µg/mL) were determined by the MTT assay results. Cells were harvested for further processing of Western blotting or RT-qPCR.

**Quantitative Real-Time Polymerase Chain Reaction.** The RNA of HUVECs was isolated using the miRVana kit (Thermo Fisher Scientific). Then, the complementary DNA (cDNA) was synthesized by Prime Script RT Master Mix (Thermo Fisher Scientific), and a Taqman Master mix kit (Thermo Fisher Scientific) was used to perform quantitative real-time polymerase chain reaction (qRT-PCR) on the 7500 Real Time System (Applied Biosystems, Waltham, MA, USA) using 1 µL of cDNA (corresponding to approximately 5 ng of total RNA) per 20 µL reaction. GAPDH was employed as the internal control. The PCR conditions were as follows: 95 °C for 10 min, 95 °C for 15 s, and 60 °C for 1 min for 40 cycles. The 2^−ΔΔCt^ method was used to calculate the relative expression. The primers were synthesized by Integrated DNA Technologies (Coralville, IA, USA). PCR primer sequences for each molecule are described in Supplementary Table S2.

**EV Internalization.** To assess HBM-EV internalization, HBM-EVs were labeled with the fluorescent dye Dil (Thermo Fisher Scientific) according to the manufacturer’s instructions [22]. Briefly, HBM-EVs were incubated in 10 µg/mL Dil solution at room temperature for one hour. Unbounded Dil was removed using Exosome Spin Columns MW 3000 (Thermo Fisher Scientific). Dil-labeled EVs were then incubated with HUVECs at a concentration of 50 µg/mL for 24 h. HUVECs were prepared in a 6-well plate (0.3 × 10^6^ cells/plate), and when the cells were 80% confluent, HBM-EVs were treated. After incubation, the cells were washed three times with PBS to remove unbound EVs. Internalization of the EVs was analyzed using fluorescence microscopy.

**Western Blotting.** Proteins were extracted from HUVECs using a RIPA buffer (Thermo Fisher Scientific). Protein samples (25 µg) were subjected to SDS-PAGE, transferred on a PVDF membrane and then probed with primary antibodies; phospho-NFκB p65 (1:1000, Ser536, Cell Signaling, Danvers, MA, USA) total NFκB p65 (1:1000, D14E12, Cell Signaling), TLR4 (1:200, 25, SantaCruz, Santa Cruz, CA, USA), and β-actin (1:10,000, C4, SantaCruz). Secondary antibodies (mouse: Santa Cruz; rabbit: Invitrogen, Waltham, MA, USA) were used at a dilution of 1:20,000. Protein bands were visualized with enhanced chemiluminescence, and the intensity of the bands was analyzed using Image J (National Institutes of Health, Washington DC, USA), with total NFκB and β-actin used as loading controls.

**Assessment of Mitochondrial Reactive Oxygen Species.** The effect of HBM-EVs on LPS-induced reactive oxygen species (ROS) in HUVECs was assessed by measuring mitochondrial superoxide using MitoSOX Red (Thermo Fisher Scientific) [23]. MitoSOX Red selectively accumulates in mitochondria and, upon oxidation by superoxide, forms a stable fluorescent compound. HUVECs were incubated with 10–50 µg/mL of HBM-EVs or an equivalent volume of PBS for 24 h. Afterward, 1 µg/mL of LPS was added for an additional 24 h. Cells were then incubated with 5 μM MitoSOX Red reagent for 10 min, following the manufacturer’s instructions. Live cell imaging was performed using a fluorescent microscope. Mean fluorescence intensity values were normalized to 100 cells and compared to the control group.

**Wound Healing Assay.** To evaluate the beneficial effects of HBM-EVs on the migration ability of HUVECs, a wound healing assay was performed. A straight scratch was introduced in the confluent cultures of HUVECs. After washing with PBS 3 times, cells were treated with HBM-EVs 50 µg/mL or vehicle controls. A time-lapse microscopy analysis of 48 h was performed, with pictures taken every 15 min. The capacity of migration was evaluated by comparing the time taken and the percentage of wound closure area.

**Animal Model Development.** All animal experiments followed the animal research guidelines from the National Institutes of Health and were approved by the University of Iowa Animal Care and Use Committee (Protocol Number: 4072418-002, approved 23 September 2024). Mice were housed in groups of 3–5 per cage with 12/12 light-dark cycle with lights on at 6:00 am. Room temperature was maintained at 21–23 °C. C57BL/6J male mice were fed a high-fat high-sucrose diet (HFHSD, 60 kcal % of fat) or normal chow diet (NCD) from 4 weeks of age for 18 weeks [9]. Their metabolic dysfunction was confirmed using a glucose tolerance test (GTT) and insulin tolerance test (ITT). GTT was performed after overnight fasting (approximately 14 h of fasting). Then glucose (2 mg of 10% of glucose per body mass) was injected intraperitoneally, and then blood glucose was measured from the tail tip using a Care Touch glucometer (Future Diagnostics, Joliet, IL, USA) before, and at 0, 15, 30, 60, and 120 min after glucose administration. The ITT was performed after 8 h of fasting. Insulin (0.75 IU insulin per body mass) was injected intraperitoneally (Novolin R Insulin 100 UN/mL, Novo Nordisk Inc., Plainsboro, NJ, USA). Then, blood glucose levels were measured from the tail tip as described above. After completion of the test, the mice were returned to their home cage and given free access to food and water.

**HBM-EV Administration**. HBM-EVs (10 μg/g of body weight) were administered daily intraperitoneally to both NCD- and HFHSD-fed mice for 2 weeks. The same amount of vehicle control (PBS) was administered to the control group. After 2 weeks of administration, the mesenteric and aorta were isolated, and a myograph study and H&E staining were performed.

**Myograph Study.** EC-dependent vasorelaxation was measured from the mesenteric arteries of HFHSD and NCD mice after 2 weeks of HBM-EV administration using a wire myograph (620 M, DMT, Essen, Germany) [24]. Mesenteric arteries were dissected from the mice, and ring fragments were mounted in Krebs–Henseleit buffer (118.4 mM NaCl, 4.7 mM KCl, 2.5 mM CaCl_2_, 1.2 mM KH2PO4, 1.2 mM MgSO_4_, 25 mM NaHCO_3_, 11.1 mM glucose). The organ bath was maintained at 37 °C, and the pH was adjusted using a gas mixture of 5% CO_2_ and 95% O_2_ throughout the experiment. Each ring was initially stretched to an optimal load of 2.0 mN. After a 30 min equilibration period, the viability of the arteries was tested with 70 mM KCl. This was followed by treatment with 10^−4^ M phenylephrine (PE). Then, serial treatments with acetylcholine (ACh, 10^−9^ M to 10^−4^ M) were applied to examine EC-dependent vasorelaxation. For the ex vivo study, mesenteric arteries were isolated from HFHSD-fed mice and tested on a myograph. Before PE-induced contraction, either HBM-EV (50 µg/mL) or PBS was added to the organ bath for 30 min, then ACh-induced EC-dependent vasorelaxation was assessed. HBM-EV was not removed during ACh treatment. At the end of the experiment, the arteries were treated with 10^−4^ M sodium nitroprusside (SNP) to assess smooth muscle function. The percentage of relaxation was compared between groups.

**Hematoxylin and Eosin (H&E) Staining of Arterial Tissue.** The 2nd branch of mesenteric arteries and the aorta were fixed in 4% PFA, paraffin-embedded, and sectioned at 5 μm. Sections were deparaffinized, rehydrated, and stained with hematoxylin and eosin, then dehydrated and cover slipped. Tissue morphology was examined under a light microscope.

**Statistical Analysis.** Experimental measures between groups were compared by the Mann–Whitney U test with a statistical threshold of *p* = 0.05 using GraphPad Prism 7 (GraphPad Software, Inc., La Jolla, CA, USA). All plots were generated using GraphPad Prism 7 (GraphPad Software, Inc.). *p* < 0.05 was considered to indicate a statistically significant difference.

## 3. Results

**Figure 1:**
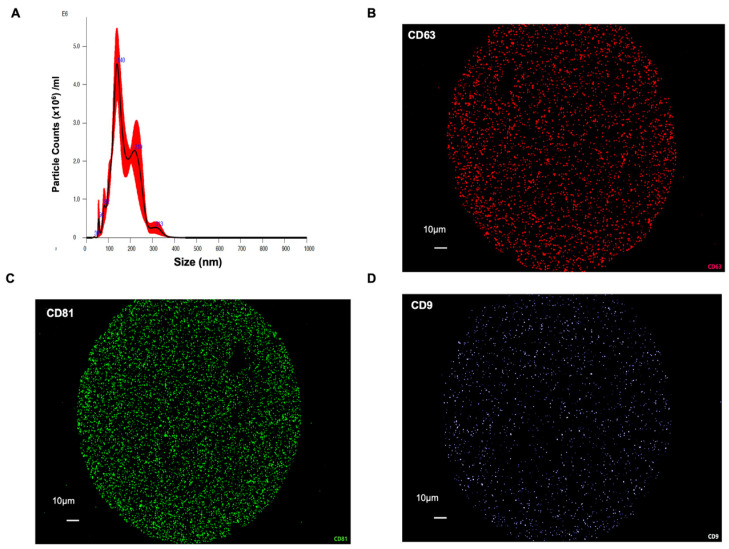
**Isolation and characterization of EVs from human breast milk.** (**A**) Nanoparticle tracking analysis (NTA) showed the size distribution and concentration of isolated HBM-EVs, with the majority of particles ranging between 100 and 200 nm, consistent with the typical size of small EVs. (**B**–**D**) Immunofluorescence-based nanoparticle tracking confirmed the presence of canonical EV markers CD63 (red), CD81 (green), and CD9 (light blue), indicating the successful enrichment of HBM-EVs.

**Isolation and characterization of HBM-EVs.** Human breast milk (HBM) was collected from 15 lactating participants; all samples were mature milk (≥19 days postpartum; mean ± SD, 28.6 ± 8.9 days). Seven donors reported no medication use; the remainder reported occasional use of dietary supplements or common over-the-counter products. None reported hormonal contraception at the time of collection (details in Supplementary Table S1). HBM-EVs were isolated by differential centrifugation coupled with ultracentrifugation as previously described [19]. Following isolation, HBM-EVs were analyzed using Nanoparticle tracking analysis (NTA), which highlighted major peaks at 145 and 135 nm (Figure 1A). The NTA also demonstrated the presence of EVs larger than 150 nm, suggesting that the isolated fractions have a heterogeneous mixture of EVs. Then HBM-EVs were characterized based on surface expression of canonical EV markers CD9, CD63, and CD81 by measuring the expression of general EV markers using fluorescence antibodies (Figure 1B–D). We also tested 37 surface epitopes of HBM-EVs. Besides CD9, CD63, and CD81, CD326 was highly expressed, which demonstrates that the majority of HBM-EVs are derived from mammary epithelial cells (Supplementary Figure S1).

**Figure 2:**
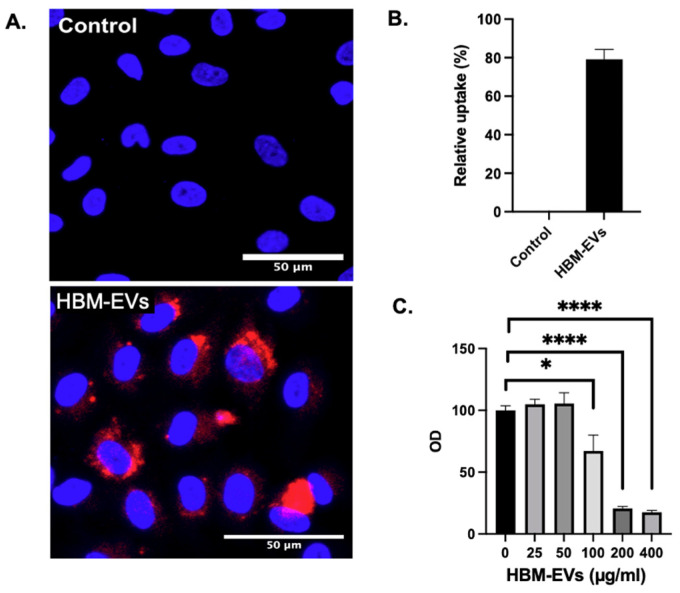
**Internalization of HBM-EVs to HUVECs.** HBM-EV internalization was assessed in HUVECs treated with 50 µg/mL of DiI-labeled HBM-EVs (red). After 24 h, the cells were fixed and stained for nuclei (blue). (**A**) shows a representative image, and (**B**) displays a quantification graph (*n* = 4). (**C**) MTT assay was performed with different concentrations of HBM-EVs to assess the cell viability of HUVECs with HBM-EVs. Based on the result, we determined to test 50 µg/mL of HBM-EVs to treat HUVECs. * *p* < 0.05, **** *p* < 0.0001 compared to HBM-EV 0 µg/mL treatment group.

**Uptake and internalization of HBM-EVs by endothelial cells.** To examine whether HBM-EVs will be taken up by ECs, HBM-EVs were labeled with Dil stain and applied to the HUVECs culture. Using confocal microscopy, we observed that HBM-EVs were efficiently internalized in HUVECs (Figure 2A). Four different HBM-EV samples from different donors were tested, with an average uptake percentage of 80 ± 5.2% (Figure 2B). These data indicate that HBM-EVs are potentially an efficient delivery vehicle to human ECs. As the only bionormal nanovesicle in the milk, HBM-EVs are predicted to be safer and less toxic. To test this hypothesis, we exposed primary HUVECs to the different concentrations of HBM-EVs and 48 h later examined their cell viability using MTT assay. We tested five different HBM-EV samples with six different concentrations. Following an overnight incubation with HBM-EVs, HUVECs showed a dose-dependent cytotoxicity to HBM-EVs. As shown in Figure 2C, the cell viability of HUVECs was not altered by HBM-EV treatment at concentrations of 0, 25, and 50 μg/mL. However, the concentration of HBM-EVs at 100, 200, and 400 μg/mL significantly reduces the cell viability.

**Figure 3:**
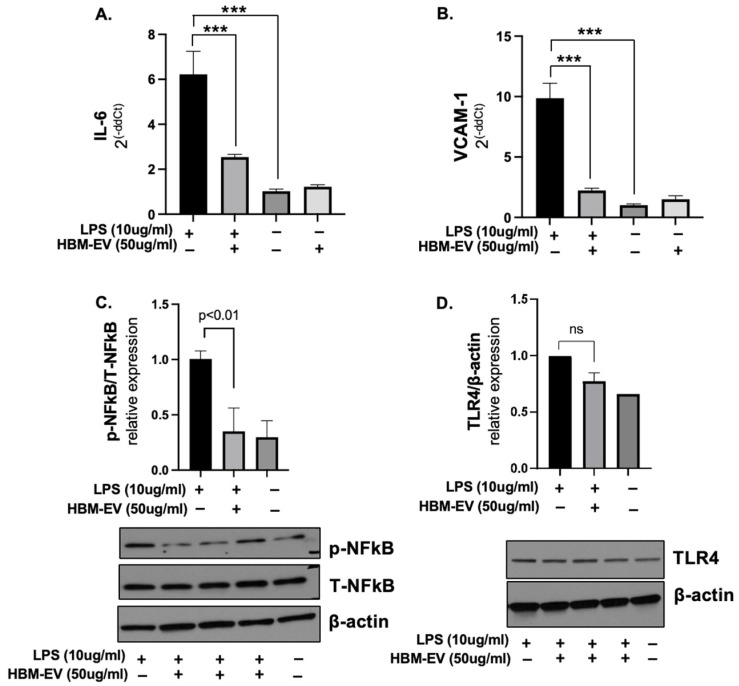
**Reduced inflammatory gene expression by HBM-EV pre-treatment in LPS-treated HUVECs.** HUVECs were treated with HBM-EVs (50 µg/mL) for 24 h, followed by the addition of LPS (10 µg/mL). After an additional 24 h, inflammatory gene expression, including IL-6 and VCAM-1, was analyzed using qPCR (**A**,**B**), and protein levels of phospho-NFkB and TLR4 were assessed by Western blotting (**C**,**D**). Each experiment was repeated three times with different HBM-EV samples (*n* = 5). Data are presented as mean ± SE. *** *p* < 0.001 and ns; no significance.

**Anti-inflammatory effect of HBM-EVs in endothelial cells.** One of the important links between endothelial dysfunction and pro-inflammatory endothelial activation is the intrinsic capacity of activated vascular endothelium to synthesize and secrete chemokines, such as interleukin-6 (IL-6). To investigate the anti-inflammatory effect of HBM-EVs, we measured these pro-inflammatory cytokines in LPS-activated HUVECs. We assessed the expression levels of IL-6 and VCAM-1 after treatment with LPS in the presence or absence of HBM-EVs. These are key mediators of endothelial inflammation and dysfunction [25]. The results revealed a significant reduction in IL-6 and VCAM-1 expression with a pre-treatment 50 µg/mL dose of HBM-EVs (Figure 3A–C). Upon LPS binding, TLR4 activates signaling pathways, including NFκB, which drive the production of inflammatory cytokines. Upon stimulation with LPS for 24 h, the phosphorylation of NFκB at Ser536 in HUVECs was noticeably enhanced. We utilized a Western blot to examine the levels of TLR4 and the phosphorylation of NF-kB. The results demonstrated that the levels of phospho-NFkB elevated by LPS treatment were significantly reduced with HBM-EV treatment (Figure 3D). However, TLR4 expression was not statistically significant. These data illustrate that HBM-EVs can abate inflammatory responses via the NFκB signaling pathway in LPS-induced HUVECs.

**Figure 4:**
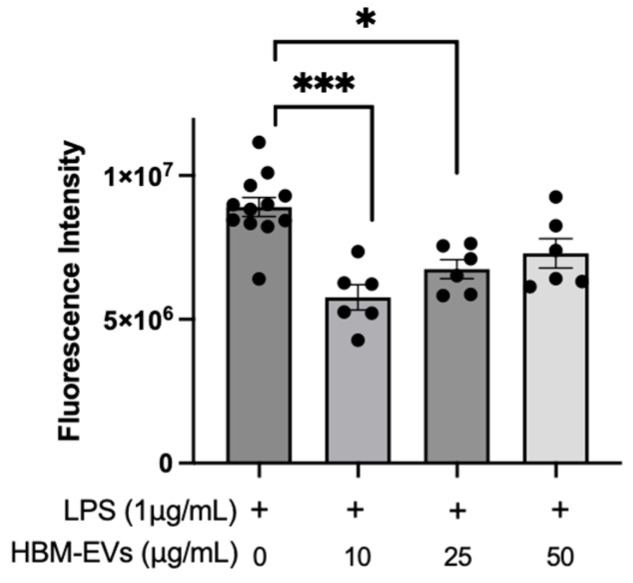
**Reduced oxidative stress in HBM-EV-treated HUVECs**. HUVECs were incubated with PBS or HBM-EVs (10–50 µg/mL, from three different donor duplicates) for 24 h, followed by a 24 h LPS treatment. Afterward, cells were incubated with MitoSOX, and fluorescence intensity was measured to quantify mitochondrial ROS production. * *p* < 0.05 and *** *p* < 0.001.

**Suppression of mitochondrial ROS by HBM-EVs in LPS-stimulated endothelial cells.** To investigate whether the anti-inflammatory effects of HBM-EVs were associated with reduced ROS levels, we measured mitochondrial ROS using MitoSOX. As shown in Figure 4, after incubation with LPS for 24 h, the fluorescence intensity remarkably increased, suggesting an excessive level of mitochondrial ROS. Pre-treatment of HUVECs with HBM-EVs 10–50 µg/mL significantly suppressed the level of mitochondrial ROS in HUVECs imposed by LPS (Figure 4). Our results indicated that HBM-EVs could attenuate mtROS generation in HUVECs.

**Figure 5:**
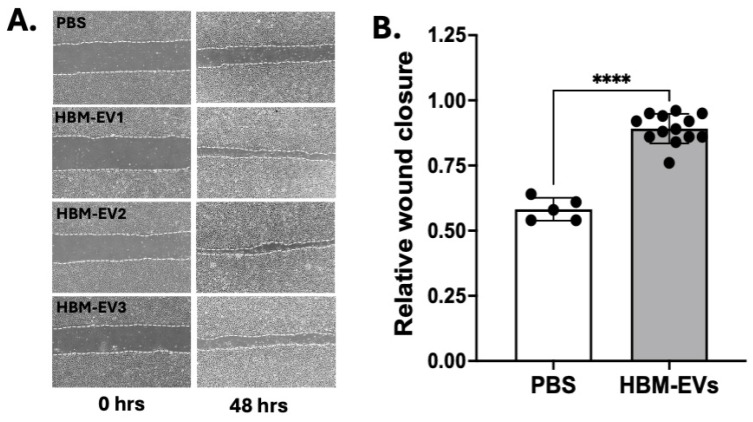
**Enhanced angiogenesis in HBM-EV-treated HUVECs.** To evaluate the beneficial effects of HBM-EVs on the migration ability of HUVECs, a wound healing assay was performed. A time-lapse microscopy analysis of 48 h was performed, with pictures taken every 15 min. Representative images (**A**) were taken before treatment and 48 h after treatment with PBS (vehicle control) or HBM-EVs (50 µg/mL, HBM-EV1–HBM-EV3 refer to HBM-EVs isolated from three different milk donors. The capacity of migration was evaluated by comparing the percentage of wound closure area at 48 h time point (**B**), *n* = 5–13. **** *p* < 0.0001.

**Enhanced wound healing of endothelial monolayers by HBM-EVs.** Next, we sought to determine whether HBM-EVs possessed bioactivity in vitro in scratch-wound assays. HUVECs were cultured in monolayers to confluency, then mechanically wounded with a pipette tip, rinsed, and provided with fresh media with either vehicle control PBS or HBM-EVs (50 μg/mL). An image of the initial scratch area (post-wound) for each group is taken and compared to the final scratch area for each group. Cells treated with HBM-EVs showed qualitatively and statistically significant (*p* < 0.0001) increases in the rate of closure of scratch wounds relative to PBS controls (Figure 5). This suggests that HBM-EVs may have a potential role in enhancing angiogenesis in ECs.

**Figure 6:**
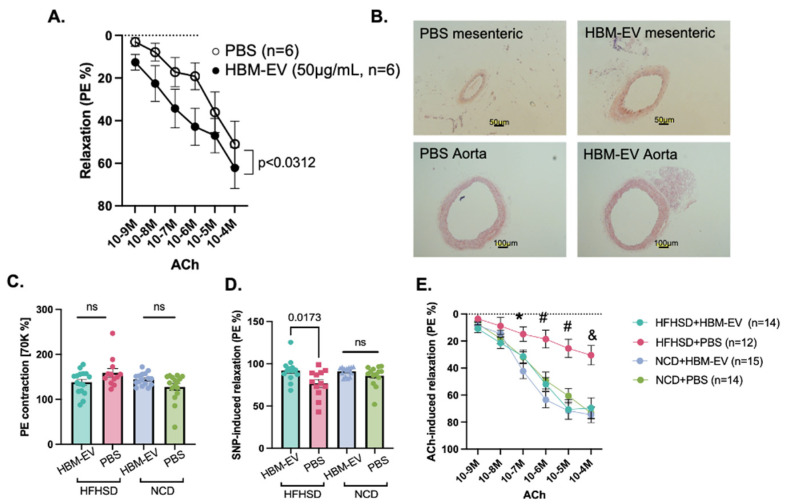
**HBM-EVs improved EC-dependent vasorelaxation in mesenteric arteries of obese mice.** (**A**) Mesenteric arteries were isolated from high-fat high sucrose diet (HFHSD)-fed mice and mounted on a wire myograph. Endothelial-dependent vasorelaxation was measured in mesenteric arteries with or without HBM-EV pre-incubation (50 µg/mL), followed by cumulative acetylcholine (ACh) stimulation after phenylephrine (10^−4^ M)-induced preconstruction. Six different HBM-EV samples from six individual donors were tested on arteries from six different mice. (**B**) After intraperitoneal (IP) administration of either PBS or HBM-EVs (10 µg/g) daily for two weeks to HFHSD-fed mice (*n* = 5 each group) and normal chow diet (NCD)-fed mice (*n* = 5 each group), aorta and mesenteric arteries were collected for hematoxylin and eosin (H&E) staining, showing that the mesenteric artery diameter was larger in HBM-EV-treated mice compared to PBS-treated controls. In mesenteric arteries, PE-induced contraction was not significantly altered by HBM-EV treatment (**C**), whereas SNP-induced vasorelaxation was improved in obese mice (**D**). Importantly, impaired EC-dependent vasorelaxation in obese mice was significantly restored following HBM-EV treatment (**E**). * *p* < 0.05, # *p* < 0.01, and & *p* < 0.001 (HFHSD + PBS vs. HFHSD + HBM-EVs). ns, not significant. Data are presented as mean ± SE.

**Improved EC-dependent vasorelaxation by HBM-EVs in diet-induced obese mice.** We also investigated the effects of HBM-EVs on EC dysfunction in animal models. Diet-induced obese mice are well known to exhibit impaired EC function [26,27]. Therefore, we assessed the impact of HBM-EVs on EC-dependent vasorelaxation in these mice through both ex vivo and in vivo studies using a myograph system. Metabolic impairment in these mice was confirmed via glucose tolerance tests (GTT) and insulin tolerance tests (ITT) (Supplementary Figure S2). For the ex vivo study, we incubated isolated mesenteric arteries of HFHSD-fed mice with PBS or HBM-EVs (50 µg/mL) for 30 min, then, EC-dependent vasorelaxation was evaluated (Figure 6A). ACh-induced vasorelaxation was significantly improved in the HBM-EV-treated group (*p* < 0.0312). For the in vivo study, we administered HBM-EVs (10 µg/g of body weight/day) or PBS intraperitoneally to normal chow diet (NCD)-fed mice and HFHSD-fed mice for 2 weeks. While the morphology of the aorta was not changed, the diameter of the mesenteric artery was enlarged, suggesting that vascular remodeling occurs by HBM-EV treatment (Figure 6B). The vascular function of mesenteric arteries was also evaluated in a wire myograph. Although vascular contractility was not changed (Figure 6C), EC-independent vasorelaxation (Figure 6D) and EC-dependent vasorelaxation (Figure 6E) were significantly improved in HBM-EV-administered HFHSD mice.

## 4. Discussion

Milk EVs are known to have anti-inflammatory properties as a component of milk. They effectively reduce inflammatory gene expression in intestinal epithelial cells; however, their effects on other cell types have not been well investigated. Previous studies using bovine milk-derived EVs have demonstrated efficient uptake by endothelial cells and pro-angiogenic effects [28,29]. In this study, using human breast milk EVs (HBM-EVs), we demonstrated their beneficial role in mitigating endothelial cell dysfunction, which plays a critical role in various cardiovascular diseases (CVDs). To our knowledge, this is the first peer-reviewed report demonstrating the protective effects of HBM-EVs on endothelial cells and vascular function.

We confirmed the anti-inflammatory effects of HBM-EVs in inflamed ECs. LPS treatment activates the TLR4/NFκB signaling pathway, leading to the upregulation of pro-inflammatory genes such as IL-6 and VCAM-1, which are key mediators of vascular inflammation and endothelial dysfunction commonly associated with obesity and cardiovascular disease [30]. Pre-treatment with HBM-EVs significantly suppressed phospho-NFκB levels, likely contributing to the downregulation of IL-6 and VCAM-1 expression. These results align with our ex vivo and in vivo findings in HFHSD-fed mice, where HBM-EV treatment restored EC-dependent vasorelaxation, further supporting their vascular protective effects.

The anti-inflammatory properties of HBM-EVs are further supported by previous studies using bovine milk-derived EVs in intestinal epithelial cells [13,15]. However, our study provides direct evidence of their functional role in vascular ECs, a less explored but clinically relevant cell type in the context of cardiometabolic disease. Interestingly, TLR4 expression remained relatively unchanged following LPS stimulation. This may be attributed to the transient nature of TLR4 regulation, as receptor levels often return to baseline following early activation and are not necessarily upregulated by their own ligand [31]. Since we assessed gene and protein expression 24 h post-LPS treatment, early changes in TLR4 levels may have been missed. Together, these findings suggest that HBM-EVs attenuate vascular inflammation by modulating NFκB–dependent signaling and may serve as a promising therapeutic strategy to mitigate obesity-related endothelial dysfunction. Oxidative stress is closely linked to inflammation and plays a critical role in endothelial dysfunction. Previous studies have shown that milk-derived EVs from bovine, camel, and human sources exert protective effects against oxidative stress in intestinal epithelial and immune cells by enhancing antioxidant defenses [32,33,34]. These protective effects have been partly attributed to activation of signaling pathways such as Wnt/β-catenin, TLR4, and PI3K/AKT, and have demonstrated therapeutic potential in inflammatory conditions. In vascular ECs, oxidative stress is a key mechanism driving the development of cardiovascular diseases. Our findings extend this line of research by showing that HBM-EVs can reduce oxidative stress in inflamed ECs, highlighting their potential as a therapeutic strategy for vascular protection in cardiometabolic disease contexts. Interestingly, within the 10–50 µg/mL range, lower HBM-EV doses suppressed mitochondrial ROS more than higher doses, indicating a non-monotonic dose–response. Given the lack of standardized dosing across milk EV studies [35,36], testing a broader range of HBM-EV doses is needed to define the minimal effective dose for inflammatory and ROS endpoints in ECs.

Our data also suggest that HBM-EVs may influence mitochondrial function in ECs. Mitochondrial dysfunction is a critical determinant of EC injury progression toward apoptosis or necrosis, and is thus an emerging therapeutic target for vascular disease [37,38]. Increasing evidence has demonstrated that other types of EVs play a significant role in regulating mitochondrial function [39]. Large EVs have been shown to contain mitochondria, while small EVs can carry mitochondrial components such as proteins and mtDNA [40,41], which may influence mitochondrial activity in recipient cells. The release of mitochondrial material via EVs is often enhanced under various pathological conditions [39,42,43]. Therefore, it is unlikely that HBM-EVs derived from healthy individuals would carry a large amount of mitochondria or their fragments. However, HBM-EV cargo profiles are known to vary depending on maternal health status, such as obesity [19,44] and the stage of lactation (e.g., colostrum vs. mature milk) [45,46]. Thus, in addition to investigating whether HBM-EVs modulate mitochondrial function directly or indirectly—and elucidating the underlying cellular mechanisms—it will also be important for future studies to examine whether milk EVs derived from pathological conditions contain mitochondrial content or related components.

While milk is known to contain pro-angiogenic factors, most studies have focused on using milk-derived EVs as delivery tools rather than directly assessing their angiogenic activity. Only a few have addressed their intrinsic effects on vascular remodeling. For example, Kim et al. reported that bovine colostrum EVs enhanced re-epithelialization, angiogenesis, and matrix remodeling more effectively than mature milk EVs, although their tube formation and proliferation effects were comparable [36]. Zhang et al. also showed that bovine milk EVs reduced cardiac fibrosis and improved cardiac function, partly through enhanced angiogenesis [29]. Consistent with these findings, our study shows that HBM-EV administration increased vessel diameter and improved endothelial cell migration, indicating enhanced angiogenesis and vascular remodeling. Since angiogenesis is a key component of vascular remodeling, these processes may be mediated by overlapping molecular pathways, including nitric oxide production and PI3K/AKT signaling [47,48]. Taken together, our data support that HBM-EVs contribute to the restoration of endothelial health and vascular function. Further mechanistic studies are warranted to elucidate the signaling pathways involved.

Our study has limitations. To minimize potential confounders of HBM-EV cargo, HBM samples were collected in the morning, before breakfast, and donors using medications for chronic medical conditions were excluded [49,50,51]. However, we did not systematically record laterality (which breast was pumped) or contraceptive use (e.g., implantable contraception); these variables will be prospectively standardized and recorded in future recruitment. The EV isolation workflow followed widely used protocols at the time but did not include pre-freezing defatting and dedicated casein-micelle depletion [52,53]. In future work, we will implement the casein-depletion protocol and employ expanded EV characterization to improve the purity of the HBM-EVs in line with MISEV2023 guidelines [54]. The in vitro assays used strong inflammatory stimuli as positive controls rather than physiologic low-grade inflammation; therefore, those results should be interpreted as proof-of-concept, and we will examine lower-grade or cytokine-based stimuli in future work. Also, in vivo experiments were performed in male mice only; inclusion of females is planned to enable sex-stratified analyses and improve generalizability.

## 5. Conclusions

Our pilot study demonstrates that HBM-EVs exert strong anti-inflammatory and pro-angiogenic effects in ECs, suggesting their potential as novel therapeutics for cardiovascular diseases. Further studies are needed to elucidate the underlying cellular mechanisms and to identify the key cargo molecules responsible for the observed effects. Such knowledge will advance the development of HBM-EV-based therapeutics as a precise and effective strategy for treating cardiovascular diseases associated with endothelial dysfunction.

## Data Availability

Data is contained within the article or supplementary material.

## Supplementary Materials

The following supporting information can be downloaded at: https://www.mdpi.com/article/10.3390/nu17182953/s1, Figure S1: Expression of EV markers in HBM-EVs; Figure S2: Metabolic alterations in lean and obese mice; Table S1: Demographics of breast milk donors; Table S2: PCR primer sequences.
