# Supplementary material for: Human Breast Milk Extracellular Vesicles Mitigate Endothelial Dysfunction
Source: Nutrients. 2025 Sep 13;17(18):2953. doi: 10.3390/nu17182953 (PMC12472446; doi:10.3390/nu17182953)
Supplement: Supplementary file 1 [file nutrients-17-02953-s001.zip › nutrients-3831872-supplementary.pdf]

Supple Table S1. Demographics of breast milk donors.

	Milk donors (n=15)	
**Age (years old, mean±SD)**	31.1±3.7	
**Ethnicity**	Caucasian (14), More than one ethnicity (1)	
**Pre-pregnancy BMI**	22.6±1.9	
**Current BMI**	24.4±3.4	
**Parity**	2.4±1.2	
**Type of delivery**	Vaginal delivery	9
	C-section	2
	Missing	4
**Feeding methods**	Direct breastfeeding	Yes (*n*=11), No (*n*=4)
	Breastfeeding after pumping	Yes (*n*=4), No (*n*=11)
	Formula Feeding	Yes (*n*=2), No (*n*=13)
**Breast milk collection time after delivery (days, mean±SD)**	28.6±8.9	
**Gestational diabetes**	0	
**Pre-eclampsia**	0	
**Medication**	No medication (*n* = 7)<br>Vitamin supplements (*n*=5), Iron (*n* = 2), stool softener (*n* = 2), acid reducer (*n* = 2), anti-histamine (*n* = 2) and single instances of others include probiotic and anti-depressant.	

Supple Table S2. PCR primer sequences.

Genes	Sequences
**IL-6**	5′-GCAAGCCAGGAAGAGTCGTC-3′ (F),<br>5′-GCTGTGCTTAGTTTATTGCCAGG-3′ (R)
**VCAM-1**	5′-TTCCCTTTGTCATCCTATGCCT3-3′ (F),<br>5′-CAGTGCGTGTCGTGGAGT-3′ (R)
**GAPDH**	5′-CACTTGGAAACTGGAACC-3′ (F),<br>5′-CGGTAGATGGCCTTCTACTG-3′ (R)

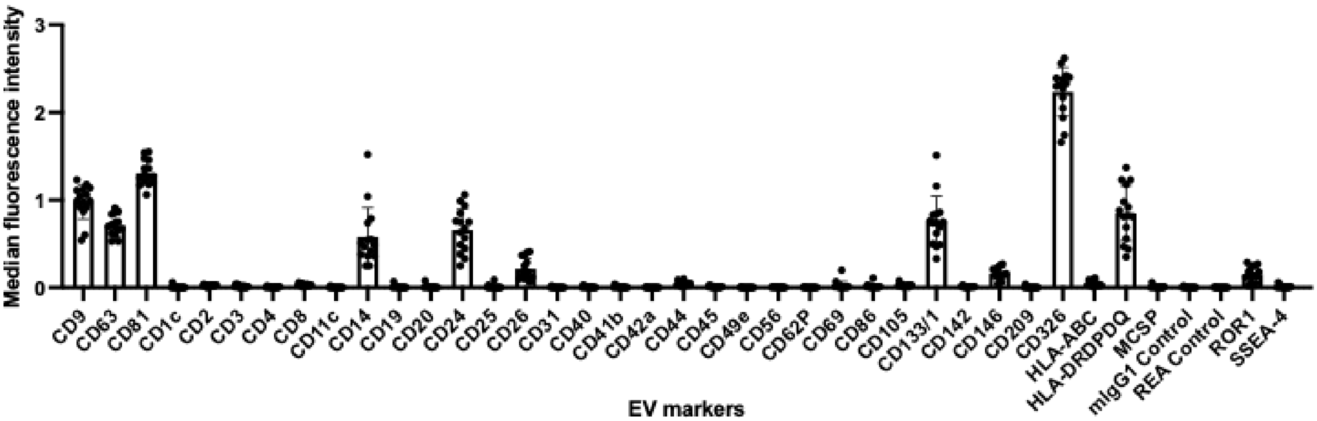

Supple Figure S1. Expression of EV markers in HBM-EVs. The median fluorescence intensity of EV markers was calculated after background correction using negative controls (*n* = 8). General EV markers, including CD9, CD63, and CD81, were highly expressed. The epithelial cell marker CD326 was also strongly expressed, indicating that the majority of bEVs are derived from mammary epithelial cells.

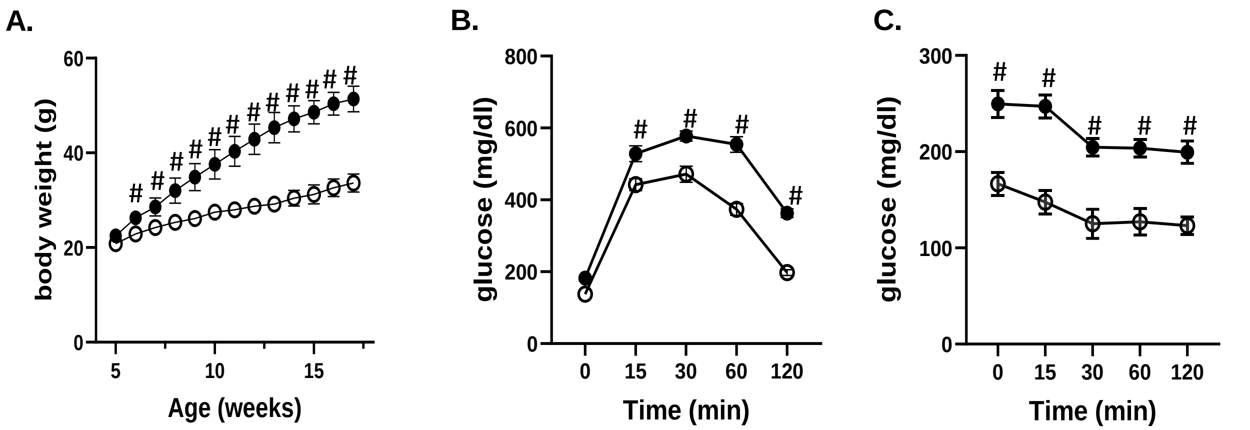

Supple Figure S2. Metabolic alterations in lean and obese mice. C57BL/6J male mice were fed either a normal chow diet (○ NCD, *n* = 6) or a high-fat diet (● HFHSD, *n* = 6) for 12 weeks. Body weight was monitored weekly (A), and glucose tolerance tests (GTT, B) and insulin tolerance tests (ITT, C) were performed at week 12. The results indicate significant metabolic impairment in HFHSD-fed mice. Data are presented as means ± SE. # $p < 0.001$.
